# Outcomes of Ipsilateral Femoral Neck and Shaft Fractures Treated With Proximal Femoral Nail Antirotation 2

**DOI:** 10.7759/cureus.18511

**Published:** 2021-10-05

**Authors:** Rajesh Rana, Himansu Behera, Sudarsan Behera, Amrit G, Madho Singh

**Affiliations:** 1 Orthopaedics, Institute of Medical Sciences and SUM Hospital, Bhubaneswar, IND; 2 Orthopaedics, All India Institute of Medical Sciences, Bhubaneswar, IND

**Keywords:** anatomical reduction, basicervical neck, cephalomedullary nail, pfna 2, ipsilateral neck and shaft fracture

## Abstract

Ipsilateral femoral neck and shaft fractures are relatively rare fractures, which most commonly occur in young adults following high-energy trauma. In most cases of such fractures, neck fracture is undisplaced and often of basicervical type. Many treatment methods have been described, but there is still no generalized consensus on the same. Cephalomedullary nails are one of the preferred modalities of treatment. A cephalomedullary nail-like proximal femoral nail antirotation 2 (PFNA 2) of recent design is being widely used currently. In this study, we present 13 cases of ipsilateral femoral neck and shaft fractures treated with PFNA 2 implants. The advantages of the PFNA 2 system include reduced blood loss, reduced operative time, and fewer fluoroscopy shots. PFNA 2 is a biomechanically better implant than many cephalomedullary implants. It provides satisfactory results in ipsilateral femoral neck and shaft fractures, especially where neck fracture is of a basicervical type. Some aspects have to be taken care of when employing PFNA 2, such as anatomical reduction, and length, angulation, and rotation of both neck and shaft.

## Introduction

Ipsilateral femoral neck and shaft fractures are rare entities, and they mainly occur due to high-velocity trauma [1,2]. They mostly occur in young adults. The incidence is around 1-9% among all femoral shaft fractures [3]. Most of the fractures have a polytrauma association [4]. The shaft of femur fractures are generally comminuted and displaced, whereas neck fractures are usually basicervical and undisplaced in 60% of the cases [5]. This indicates that most of the energy is absorbed by a femur shaft [3]. These injuries have been on the rise with the increase in high-velocity trauma cases. Fixation of both fractures should be done early with accurate reduction. Especially, neck femur fracture carries the risk of complications due to delays in surgery, such as an increased chance of avascular necrosis (AVN) in the long term [6].

Earlier, these fractures were treated by two implants addressing both the fractures separately. The neck was fixed with a dynamic hip screw (DHS) or cannulated cancellous screw, and the shaft was fixed with a retrograde nail [7]. This method had disadvantages such as increased operative time and increased blood loss [8]. The advantage of this method was a more accurate reduction of displaced neck fractures. Cephalomedullary nail is used afterward, which has lower operative time and blood loss [8]. One more advantage of this nail is that there is no stress riser zone, unlike double-implant cases [9]. Among different cephalomedullary nails, the proximal femoral nail antirotation 2 (PFNA 2) system use has increased in recent years in proximal femur fractures [10]. The PFNA 2 system has an advantage over other cephalomedullary nails with less operative time, fewer complications, and less blood loss [11]. PFNA 2 is designed for pertrochanteric fractures and basicervical neck fractures [12]. Most ipsilateral neck and shaft fracture cases have basicervical fractures [13]. Accurate reduction and maintaining reduction of neck fracture during nail placement is vital in PFNA 2. Maintaining rotational stability of the head and neck during surgery is also technically demanding. Only a few studies have discussed the results of PFNA 2 use in ipsilateral femoral neck and shaft fractures. Our study evaluates the functional and radiological outcomes of cases treated with PFNA 2.

## Materials and methods

Between 2018 and 2021, 13 cases of ipsilateral femoral neck and shaft fractures were operated on and treated with PFNA 2 at our institution. Our study analyzes a series of cases and evaluates their outcomes. We included all patients treated with cephalomedullary nail PFNA 2 as a single implant for both fractures. All patients were less than 60 years in age. Other peritrochanteric fractures, iatrogenic femoral neck fractures sustained while doing femoral shaft fracture nailing cases, patients treated with another type of cephalomedullary nails, and compound fractures of the femur were excluded. We excluded patients treated with another type of cephalomedullary nails. Compound fractures of the femur were also excluded. PFNA 2 was used as an implant in all cases, which is an intramedullary nailing system commonly used for peritrochanteric fractures.

**Figure 1:**
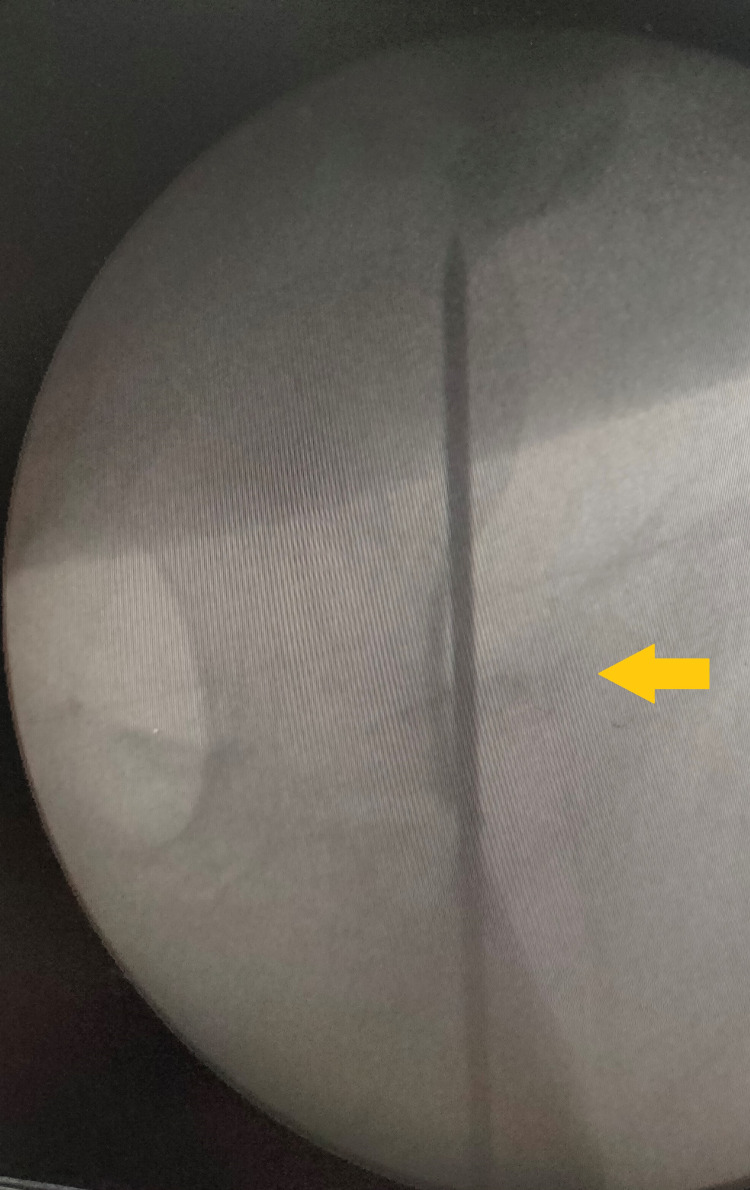
Neck reduction maintained by K-wire inserted anteriorly (arrow)

All the patients were evaluated clinically and radiologically, and other system injuries were assessed. We operated the patients in a supine position on a fracture table. Traction was applied from the foot by wrapping it in the boot of the fracture table. The reduction was tried in the traction table, and it was checked with fluoroscopy. Head and neck femur reduction was checked in anterior and posterior view and in lateral view. Most of the cases were operated on either in a close manner or with the mini-open approach at the shaft femur fracture level. After proper reduction of a neck fracture, one or two K-wires were inserted anteriorly to hold the reduction (Figure 1).

**Figure 2:**
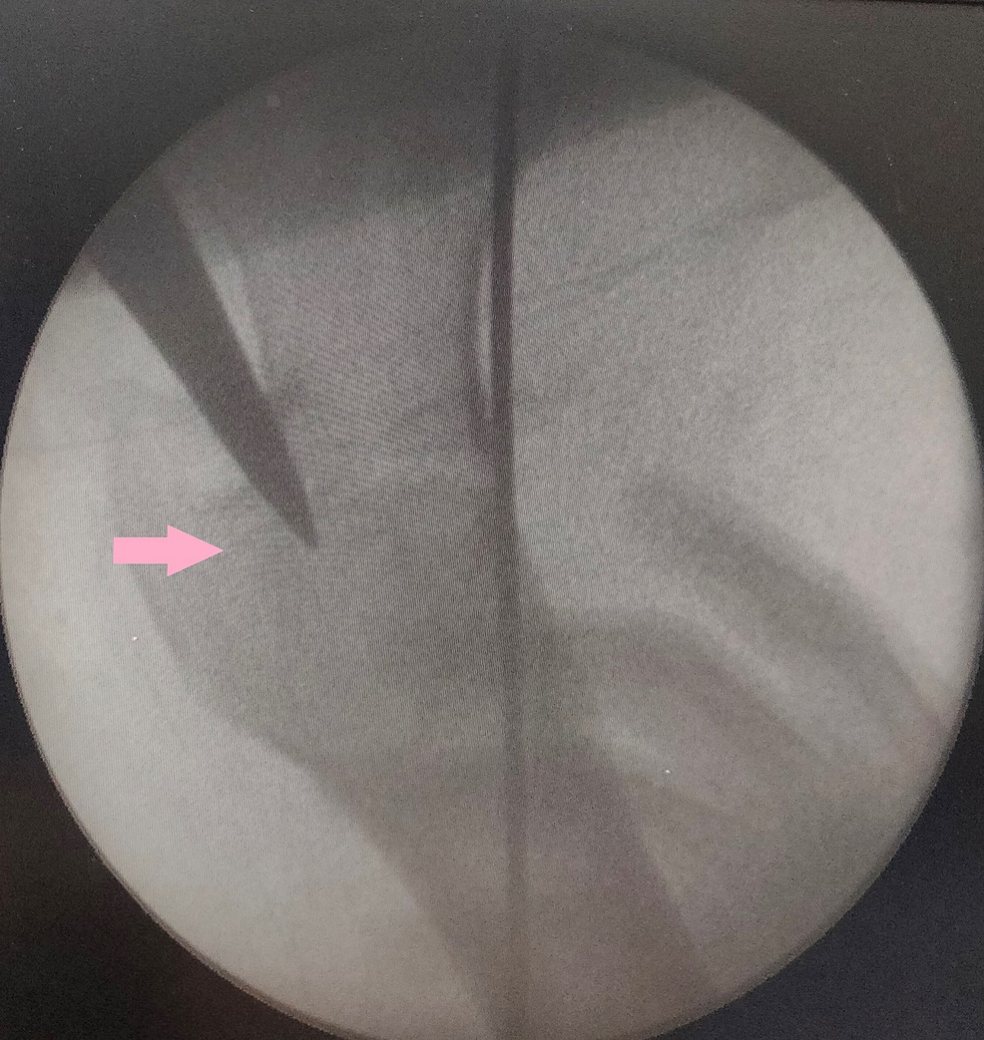
Entry for the proximal femoral nail with K-wire in place (arrow)

The position of the K-wire was checked under fluoroscopy so that it would not come in the way of the blade screw or nail (Figure 2).

Sometimes, a Steinmann pin was used above the lesser trochanter to correct the rotational alignment and reduce the proximal femur fragment.

The skin incision was done at about 5 cm proximal to the greater trochanter in the line of the femur shaft under fluoroscopy guidance after sound reduction. A guidewire was given through the entry point and was centered in anteroposterior (AP) and lateral views. The reduction of neck fracture was facilitated mainly by traction and internal rotation. After the guidewire entry, the medullary canal was prepared by reaming with 1 to 1.5-mm larger size of the chosen nail. Reaming and reduction should be adequate before nail entry to prevent forceful manipulation of the nail, which may displace the fracture, especially the neck fracture. The nail was inserted in a twisting motion. Passage across the fracture site was checked under fluoroscopy to prevent misalignment. During the nail insertion procedure, K-wires present in the neck prevented displacement and neck reduction loss. After the complete insertion of the nail, the guidewire for the blade plate was inserted into the head. The wire is preferred in the slightly lower half of the neck in the AP view. The guidewire was inserted up to 5-mm level from the joint. Drilling was done over the guidewire at 10-15 mm shorter than the tip. Most of our patients were in the younger age group and hence required full drilling length. Blade plate was given over the guidewire under fluoroscopy guidance. Then, the blade plate was locked after checking the gaps closed. The distal femur alignment was checked, and some of the rotational alignment was corrected by internal and external rotation. Then, distal locking of the nail was done by two screws.

**Figure 3:**
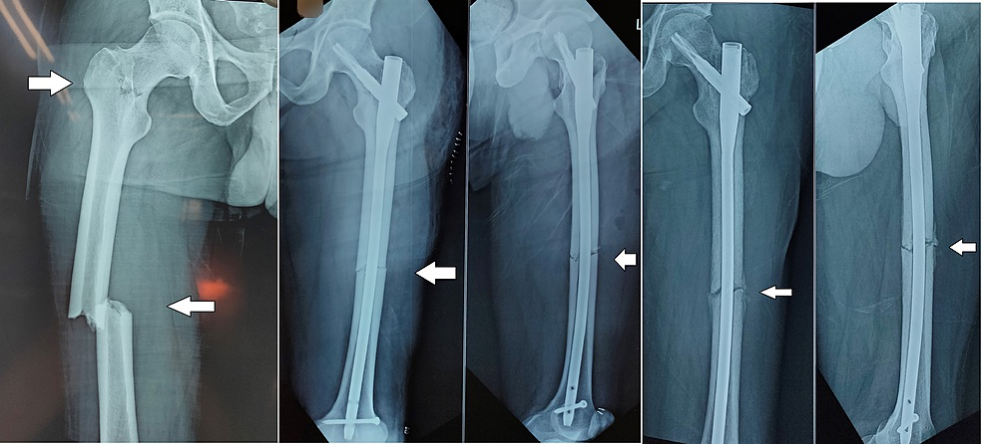
Radiograph - case 1

**Figure 4:**
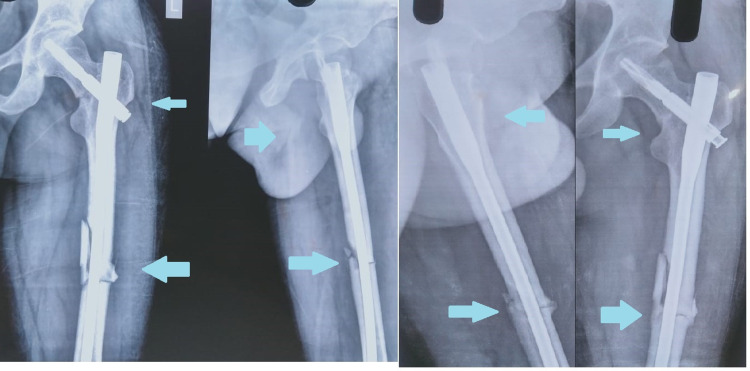
Radiograph - case 2

The patients were not allowed to weight-bear for six to eight weeks postoperatively. After that, partial weight-bearing was allowed, and radiographs were taken (Figures 3, 4). After fracture healing, full weight-bearing was gradually allowed. Radiographic follow-up was performed every six weeks for the first three months, then at every three months for 12 months. After that, it was done once a year. All patients were followed up till they achieved radiographic union.

## Results

Out of 13 operated cases, eight (62%) were male, and five (38%) were female. All patients were in the age group of 20-55 years with a mean age of 30 years (Table 1).

**Table 1:** Master chart showing demographic, functional, and radiological features AVN: avascular necrosis

Serial number	Sex	Age (years)	Mechanism of injury	Operative time (minutes)	Time to surgery (days)	Associated injury	Neck of femur fracture type	Blood loss (ml)	Follow-up (months)	Neck union (months)	Femur union (months)	Outcome (Harris hip score)	Complication
1	Male	54	Road traffic accident	65	3	Head injury	Garden 1/besicervical	250	15	3	5	Good	None
2	Male	45	Road traffic accident	67	5	None	Garden 2/besicervical	200	18	4	4	Fair	None
3	Male	23	Fall from height	75	4	Calcaneus fracture	Garden 2/besicervical	168	20	6	5	Fair	Delayed union of the femur
4	Female	44	Road traffic accident	71	6	Hemothorax	Garden 3/besicervical	175	22	5	6	Good	None
5	Male	35	Road traffic accident	78	7	None	Garden 2/besicervical	260	24	4	7	Fair	Superficial infection
6	Female	32	Road traffic accident	74	4	None	Garden 2/transcervical	210	12	5	4	Good	None
7	Male	29	Road traffic accident	67	3	None	Garden 2/besicervical	230	10	3	6	Fair	None
8	Male	33	Fall from height	75	2	None	Garden 2/transcervical	200	9	4	5	Good	None
9	Male	38	Road traffic accident	77	3	None	Garden 2/besicervical	190	17	3	8	Good	None
10	Female	41	Road traffic accident	79	2	None	Garden 3/transicervical	215	23	5	5	Poor	Nonunion, AVN head
11	Female	29	Road traffic accident	80	4	None	Garden 2/besicervical	250	18	4	6	Good	None
12	Male	51	Road traffic accident	90	5	Abdominal injury	Garden 2/besicervical	180	Lost to follow-up			Fair	None
13	Female	41	Road traffic accident	68	3	None	Garden 2/besicervical	200	16	5	5	Good	None

All patients had injuries due to road traffic accidents (85%) except two (15%) who had a fall history. The average operative time was 74 minutes, (range: 65-90 minutes). The average blood loss was 209 ml. All patients were followed up for an average period of 17 months (range: 9-25 months). One patient was lost to follow-up after five months. One case was garden type 1 fracture, two cases were garden type 2, and all other cases were garden type 3.

According to anatomical classification based on location, three fractures were transcervical, and the rest were basicervical types. Among femur fractures, six were simple fractures, four were wedge fractures, and three were comminuted fractures. All cases had sound anatomical reduction before fixation. All cases were provisionally fixed with K-wire for neck reduction before nail insertion (Figure 2). All reductions were facilitated and maintained by a traction table. Out of 13 patients, four patients had other systemic injuries like chest, abdomen, head injury. The average period from injury to operation was four days (range: two to seven days). All cases achieved union with good functional outcomes except for two patients. Out of these two cases, one had a nonunion of the neck of the femur, which led to AVN of the head. Total hip replacement was done for that later. The second patient had delayed union of shaft of the femur. One case had a superficial infection over the femur reduction incision site, which healed uneventfully. All cases were fixed with PFNA 2 system. The tip apex distance (TAD) of the screw tip was 18.2 mm (range: 13.5 to 24.6 mm) (Case 1). Radiographs of two cases are presented below (Figures 3, 4).

## Discussion

There is no generalized consensus on the treatment of ipsilateral femoral neck and shaft fractures [8]. Surgeons are often confused as to which fracture should be addressed first and which implant to be used. Most of the time, surgeons have to choose between a single implant like a cephalomedullary or a double implant like the DHS or a cancellous screw for the neck fracture and retrograde nailing for the shaft fracture [13]. The reason behind this is the rarity of these fractures and the many methods of treatment available. Most of the fractures are due to high-energy trauma in relatively younger people and often polytrauma [14]. Many techniques have been described in the literature, but PFNA 2 is a relatively newer implant used for these fractures. PFNA 2 is most commonly used for peritrochanteric fractures. Kein Tung et al. have described good results in their series with PFNA 2 implant [15]. It is a relatively strong implant with good rotational stability [16]. Earlier, double implants were preferred for ipsilateral neck and shaft fractures with the priority given to neck fracture reduction. Reduction of displaced neck fracture is relatively difficult with cephalomedullary nails like PFNA 2. Under image intensifier guidance, it can be done using the above method. Reduction of the neck is maintained by temporary K-wire fixation. Placing K-wire for maintaining neck and head area reduction is difficult because of the large proximal part of PFNA 2. In our cases, we found that it could be given anteriorly or posteriorly without hampering the nail passage.

In most cases, we placed the wire anteriorly so that, if required, a nail and blade screw could be given at the center or posterior inferiorly. Undisplaced and minimally displaced basicervical neck fractures maintain the reduction on the traction table. Optimal neck reduction and basicervical neck fracture type cases achieve good results with PFNA 2. Transcervical intracapsular cases are relatively difficult to manage with PFNA 2 because of the chance of rotation while giving derotation helical blade screw. Out of the three transcervical necks of femur fracture cases in our series, we encountered one complication of nonunion, which subsequently led to AVN of the head. The percentage of AVN in our study is in line with the previous studies, which is roughly 3% [6]. It was treated with a total hip replacement later. PFNA 2 is associated with another complication as reported in many studies: that of the medial cutout [17]. This can be prevented by ensuring optimal TAD. In our study, we did not face this complication.

In these types of fractures, femur shaft fracture is often displaced and comminuted [18]. According to the Winquist classification, these fall under grade 3 and 4 categories. This occurs because most of the traumatic force is dissipated at the shaft and less amount at the neck. A neck fracture is often undisplaced and often missed in 6-22% of cases [19]. In our study, the pattern was the same. Highly comminuted and displaced fractures increase the chance of delayed union. A few cases required open reduction for accurate reduction. All fractures achieved union at the 12-month follow-up.

The most commonly used cephalomedullary nail for these types of fractures is PFNA 2. Compared to a regular PFN implant, PFNA 2 has certain advantages like less blood loss, reduced number of fluoroscopy shots, reduced duration of surgery, and shorter hospital stay [13]. If the TAD of the neck screw is kept adequate, then the failure rate in PFNA 2 is less than that in regular PFN [20]. PFNA 2 has less complication rate in comparison to other cephalomedullary nails like PFN [11]. Implant-related complications like screw backout and cutout through bone are usually minor in PFNA 2. Huang et al. conducted a comparative study between PFNA and Recon nails [21], and they found that PFNA had better results.

## Conclusions

There is still no generalized consensus regarding the treatment of ipsilateral femoral neck and shaft fractures. With the advent of newer implants like PFNA 2, treatment strategies are getting updated regularly. Cephalomedullary implant for these types of fractures is a preferred modality of treatment. In our study, we observed that PFNA 2 has good functional outcomes, especially with respect to basicervical neck fractures with the shaft of femur fractures, with less complication. But our research has some limitations, such as the small sample size due to the rarity of these fractures, as well as the absence of a comparison group. Further, larger randomized clinical trials are required for validating our findings, which may be quite challenging for such rare types of fractures.
